# From observation to optimization: behavioral metrics that matter in KPI based home cage monitoring

**DOI:** 10.3389/fnbeh.2026.1694689

**Published:** 2026-03-02

**Authors:** Steven R. Talbot, Fabrizio Scorrano, Stefano Gaburro, Pierre Lainee, Marcel M. van Gaalen

**Affiliations:** 1 Institute for Laboratory Animal Science, Hannover Medical School, Hannover, Germany; 2 Comparative Medicine, Novartis International AG, Basel, Switzerland; 3 Tecniplast S.p.A., Buguggiate, Italy; 4 Translational Models, Sanofi Research & Development, Montpellier, France; 5 Translational Sciences, Evotec International GmbH, Göttingen, Germany

**Keywords:** behavioral neuroscience, digital biomarkers, home cage monitoring, key performance indicators, return on investment

## Abstract

Most *in vivo* scientists would agree that digital biomarkers collected via home-cage monitoring generate valuable data. However, few can tell precisely how valuable. The gap between enthusiasm and evidence has slowed the adoption of digital biomarkers in preclinical research. This framework paper addresses that gap by providing explicit key performance indicators (KPIs), organized into scientific, operational, welfare, and financial categories. We show how return-on-investment calculations differ across pharmaceutical companies, contract research organizations (CROs), and academic institutions. Furthermore, we demonstrate the approach through a worked example in an Amyotrophic Lateral Sclerosis (ALS) mouse model that reduces full-time equivalent (FTE) requirements by half. When successfully integrated, digital biomarkers can generate richer datasets, reduce the number of animals, improve welfare, and enhance translational value. However, successful implementation requires clear performance metrics to justify investment and measure success. We also discuss what these technologies cannot do, because understanding limitations matters as much as understanding benefits.

## Introduction

The paradox of home cage monitoring is that the technology to generate digital biomarkers exists and its benefits are clear, yet most animal facilities remain unequipped to use it. The challenge is not scientific skepticism, but the inability to express value in terms that resonate with decision makers. Clinical research has driven explosive growth in digital biomarker data over the past decade (Macias Alonso et al., 2024). Wearables, sensors, and continuous monitoring devices now generate streams of information that were unimaginable a generation ago. The convergence of artificial intelligence and machine learning with these expanding datasets promises applications that we are only beginning to explore (Huang et al., 2023). In clinical practice, digital biomarkers can alert practitioners to anomalous patterns, provide objective continuous evaluations, and establish endpoints for trials assessing disease-modifying treatments (Kourtis et al., 2019).

The preclinical field is beginning to embrace these approaches. Home cage monitoring systems now enable continuous, longitudinal, noninvasive assessment of an animal’s behavior within their living environment. By recording data around the clock, these systems generate volumes of information that data scientists can analyze using computational methods to derive and validate preclinical digital biomarkers. The potential is significant: better understanding of pathologies, support for drug discovery, increased reproducibility, and meaningful improvements in animal welfare through the 3Rs principles of Reduce, Refine, and Replace (Voikar and Gaburro, 2020).

Yet potential alone is not enough. Equipping an animal facility requires substantial investment. Implementation requires collaboration among *in vivo* scientists, data scientists, IT professionals, and biostatisticians. The question that halts most projects is straightforward: what is the return on investment (ROI)? The honest answer has been that we do not yet know how to calculate it. This paper offers a framework to address that question and delivers three key components. First, we provide explicit KPI organized into scientific, operational, welfare, and financial categories. Not vague concepts like efficiency or impact, but specific metrics with formulas, measurement methods, and target benchmarks. Second, guidance on how ROI calculations differ across organizational contexts. Pharmaceutical companies, CROs, and academic institutions have different priorities. Their KPIs should reflect that. Third, a worked example demonstrating how these metrics apply to a real study design in an ALS mouse model.

This paper aims to provide a clear and practical framework for evaluating the value of digital biomarkers in preclinical research, outlining KPIs, organizational considerations, and a real-world application example to support informed investment and successful implementation.

## Digital biomarkers: definitions and classification

### What are digital biomarkers?

Ask 10 researchers to define a digital biomarker, and you may get 12 answers. A recent review identified over 120 published definitions (Macias Alonso et al., 2024). This lack of consensus creates confusion but also opportunity. For this framework, we define digital biomarkers as *quantifiable physiological or behavioral measures collected through digital devices that indicate biological processes, pathological states, or responses to interventions*. In preclinical work, these measures are primarily derived from automated home-cage monitoring systems that continuously capture data without disturbing the animals.

What makes a digital biomarker translational? We propose that translational digital biomarkers involve continuous recordings collected over days or weeks that provide insight across species, including humans. For example, a locomotor activity pattern that predicts disease progression in mice and aligns with wearable sensor data in patients would qualify as a translational digital biomarker.

### Applications across laboratory animal science

Consider locomotor activity. This single metric can support welfare teams by identifying cages that need attention (Collins et al., 2025) and assist veterinarians and ethics committees in evaluating environmental changes or project protocols. But it can also help researchers to phenotype transgenic strains, detect adverse drug effects early, or serve as an efficacy marker in neuroscience or oncology studies. One behavioral metric, multiple applications, different stakeholders.

This breadth matters for implementation decisions. A system optimized for welfare monitoring may not meet the needs of a drug discovery program. Understanding your primary application shapes which KPIs matter most.

### Classification by clinical maturity

Not all digital biomarkers are created equal. Some build on decades of validation. Others remain exploratory. We find it helpful to classify them by maturity:

*Established digital biomarkers* enhance or expedite accepted practices. Heart rate monitoring is the classic example. The measurement is validated. The clinical applications are known. The digital version simply does it better, faster, or more continuously than traditional approaches.

*Emerging digital biomarkers* represent two scenarios. A novel measurement approach applied to an established clinical domain, such as smartphone gait analysis for Parkinson’s disease (Abou et al., 2021). Or an established measurement applied to a new domain, such as continuous activity monitoring for mood disorders (e.g., Shui et al., 2025).

*Novel digital biomarkers* pair new measurement approaches with new applications. Facial expression analysis for pain assessment in rodents would be an example. These carry the most uncertainty but also the most potential for breakthrough insights.

### FDA biomarker categories

The FDA’s BEST Resource provides standardized categories that apply regardless of whether a biomarker is digital: susceptibility and risk biomarkers indicate disease potential; diagnostic biomarkers detect or confirm conditions; monitoring biomarkers track status over time; prognostic biomarkers predict clinical events; predictive biomarkers identify likely responders to treatment; response biomarkers show biological effects of exposure; and safety biomarkers indicate toxicity. Similar frameworks exist internationally. The European Medicines Agency (EMA) operates a biomarker qualification program that follows comparable categorical distinctions. The International Council for Harmonization (ICH) provides guidance on biomarker development that applies across regulatory jurisdictions including Europe, Japan, and North America (ICH E16). These categories help clarify what a digital biomarker is actually measuring and facilitate regulatory acceptance across multiple markets. In the same trend, revision of the ICH S7A guidance document (referring to Safety Pharmacology) also considers including references to increased use of digital behavioral biomarkers.

## Key performance indicators for home cage monitoring

This is where most discussions of home cage monitoring go wrong: they describe benefits without quantifying them. Terms like efficiency and impact, without defining what they mean in measurable terms, are missing the point. A KPI that cannot be measured is not a KPI; it remains a fuzzy wish.

We organize KPIs into four categories: Scientific, Operational, Welfare, and Financial. Each contains specific metrics with explicit calculation methods and target benchmarks. The target values presented here are not universal standards. They represent reasonable ballpark figures derived from our collective experience across pharmaceutical, CRO, academic, and technology-provider environments. Individual organizations should calibrate these targets based on their specific operational context, research priorities, and resource constraints. This framework is based on our collective experience across pharmaceutical, CRO, academic, and technology-provider settings.

### Scientific KPIs

Scientific KPIs measure what matters to researchers: data quality, statistical power, and translational value.

*Temporal data density* counts data points per animal per day (see Table 1). Traditional approaches yield one or two daily measurements at most, for example, body weight or weekly behavioral testing. Home cage monitoring can continuously generate thousands of data points, detecting patterns invisible to snapshot methods. *Target: more than 1,000 data recorded points per animal per day for potential analyses strategies.* This threshold reflects the minimum sampling frequency needed to capture circadian patterns and detect transient behavioral events that snapshot methods miss.

**Table 1:** KPI framework summary.

Category	KPI	How to calculate	Target*
Scientific	Temporal data density	Data points/animal/day	>1,000/day
	CV reduction	(CV_conv_ − CV_HCM_)/CV_conv_	>30% reduction
	Translational concordance	Correlation with clinical data	*κ* > 0.6
Operational	FTE reduction ratio	(Hours_conv_ − Hours_HCM_)/Hours_conv_	>40% reduction
	Sample size reduction	(*N*_conv_ − *N*_HCM_)/*N*_conv_	>20% reduction
Welfare	Early detection lead time	Days before conventional detection	>2 days
Financial	Payback period	Investment/annual savings	<3 years

*Target values represent reasonable ballpark figures based on collective author experience. Individual organizations should calibrate targets to their specific operational context.

*Within-subject variability* reduction is assessed by comparing the coefficient of variation for repeated measures. Animals tested in their home environment show less measurement-induced variability than those repeatedly handled. *Target: more than 30% reduction in the* coefficient of variation *compared to conventional testing.* This figure reflects improvements observed in published comparisons between home cage and conventional testing paradigms (Voikar and Gaburro, 2020).

*Effect size* improvement measures the standardized mean difference between groups. Reduced measurement noise can translate to larger detectable effects, but this relationship is not automatic. Long-term continuous recording generates high-volume, temporally heterogeneous datasets that require sophisticated analytical pipelines to extract meaningful signals. Without appropriate statistical methods, increased data volume can paradoxically increase apparent noise. The target assumes that proper data reduction and analysis strategies are in place. *Target: more than 0.3 improvement in Cohen’s d compared to conventional testing when appropriate analytical methods are applied*.

*Translational concordance* correlates preclinical measures with clinical endpoints. This requires validation studies comparing animal outcomes with human trial data. It is the hardest KPI to achieve but potentially the most valuable. *Target: weighted Cohen’s kappa above 0.6 for clinical severity grades.* A kappa of 0.6 represents “substantial agreement” under standard interpretation scales and is a common threshold for acceptable concordance in translational studies.

### Operational KPIs

Operational KPIs quantify efficiency gains. These translate most directly into cost savings.

*The FTE reduction ratio* is calculated (Hours_conv_ minus Hours_HCM_) by Hours_conv_, expressed as a percentage. If a study traditionally requires 192 h of testing and home cage monitoring reduces that to 101 h, the FTE reduction ratio is 47%. *Target: more than 40% reduction for phenotyping studies.*

*Sample size reduction* applies the same formula to the number of animals. Reduced variability allows smaller groups to achieve equivalent statistical power. *Target: more than 20% reduction in animal numbers due to optimized statistics and procedures.*

*Time-to-decision* measures the days from study start to the actionable endpoint. Earlier detection accelerates programs. *Target: more than 15% reduction in decision timelines.*

### Welfare KPIs

Welfare KPIs matter ethically and are increasingly required by regulatory and publication requirements.

*Early detection lead time* measures the number of days between an algorithm-generated alert and conventional detection. Well-designed algorithms can identify cages needing attention before human observers notice problems (Theologidis et al., 2025; Tomanelli et al., 2024). Target: achieve early warnings at least 2 days in advance.

*Handling frequency reduction* compares handling events under conventional versus home cage protocols. Home-cage monitoring reduces animal handling, resulting in less stress. This KPI clearly has an operational aspect for animal welfare teams as well. *Target: more than 50% reduction in handling events.*

### Financial KPIs

Financial KPIs enable the ROI calculations that justify investment.

The *payback period* is calculated by dividing the total capital investment by the annual operational savings. A $200,000 system that saves $80,000 annually has a payback period of 2.5 years. *Target: less than 3 years for full deployment.* This aligns with typical capital equipment depreciation cycles in research institutions and represents a threshold that most finance departments consider acceptable for non-revenue-generating infrastructure.

*Cost per data point* is the total operational cost divided by the number of data points generated. Home cage monitoring should dramatically reduce the marginal cost of additional measurements. *Target: less than $0.01 per data point after initial investment.*

## Implementation contexts

Pharmaceutical companies and university laboratories may both want to implement home cage monitoring, but for different reasons. Their KPI priorities should, therefore, reflect that motivational difference.

### Pharmaceutical industry

For pharmaceutical companies, translational value likely drives decision-making. Can this preclinical measure predict what will happen in patients? Can we fail faster when compounds do not work, to assure investment in the most promising drug discovery programs? Can we build datasets across programs that make our discovery engine smarter over time?

The priority KPIs are translational concordance, effect size improvement, and time to decision. The “fail early” strategy is critical: stopping a drug discovery program even a few months earlier can save millions. That potential ROI justifies significant upfront investment, even when the exact value is hard to calculate prospectively.

### CROs

CROs face a different calculus. They must offer state-of-the-art technology to win business. But they also need that technology to pay for itself. Data confidentiality limits their ability to merge datasets across clients. Every investment must show a clear financial return.

The priority KPIs are the FTE reduction ratio and the payback period. A CRO that can complete studies with 40% less technician time gains a significant competitive advantage. The central issue is whether the labor savings achieved over the lifespan of a home-cage monitoring system are sufficient to justify its initial investment.

### Academic institutions

Academic institutions value a different currency, like knowledge, publications, and reputation, rather than direct financial returns. Reproducibility has become a career issue as journals and funders demand it. Animal ethics committees scrutinize 3Rs compliance. Grant applications increasingly require data sharing plans. These priorities translate into different KPIs: Coefficient of Variation reduction and reproducibility metrics, sample size reduction for 3Rs compliance, and contributions to data sharing.

The welfare KPIs matter for all categories.

## Calculating ROI

### The basic formula

ROI equals (Total Benefits minus Total Costs) divided by Total Costs, expressed as a percentage. Although theoretically simple, the challenge is to capture Total Benefits in monetary terms. How much is a 30% reduction in variability worth? The answer depends on how that reduction is leveraged.

### Cost components

*Capital expenditure*: Hardware, sensors, cameras, installation. The cost of systems such as Digital Ventilated Cages, as with other home cage monitoring solutions, rises in proportion to the number of cages being monitored.

*Computing infrastructure*: A facility with 50 monitored cages generating roughly 39 GB per hour accumulates about 340 TB annually. Cloud storage runs $10,000 to $50,000 per year, depending on redundancy requirements. GPU computing for AI analysis adds additional costs.

*Software and licensing*: Fees vary by features, cage counts, and support levels.

*Personnel*: Training, ongoing support, and data science time for developing analysis pipelines.

### Worked example: application in an ALS mouse model

ALS mouse models clearly illustrate the value proposition. Traditional assessment relies on periodic functional outcomes from assays such as grip strength tests and rotarod performance, imaging sessions, and endpoint evaluations. These methods are effective but labor-intensive and may miss subtle changes between scheduled assessments.

### Study parameters

Our example uses 40 mice, divided into 4 groups of 10, monitored from 6 to 40 weeks of age. We define one FTE as 160 working hours per month. Time estimates are based on standard operating procedures at the authors’ institutions across Hannover Medical School, Novartis, and Sanofi.

### Conventional approach

**Figure 1:**
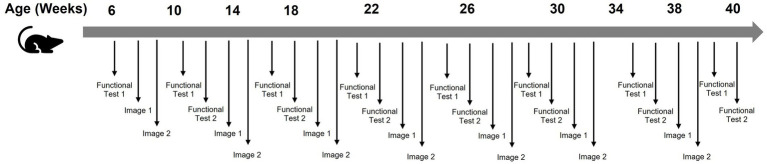
Example of ALS study without home cage monitoring.

Time estimates below include setup, calibration, animal handling, data recording, and equipment maintenance (see Figure 1). “Assessment cycle” refers to 1 month of study duration.

Functional Test 1 (grip strength): approximately 5 min per animal times 40 animals equals roughly 3.3 h per session. With testing every 2 weeks (twice per assessment cycle), plus setup and calibration overhead, this totals approximately 8 h per assessment cycle.

Functional Test 2 (rotarod): approximately 2.5 min per animal times 40 animals equals roughly 1.7 h per session. Including training trials, equipment preparation, and twice-monthly testing, this totals approximately 8 h per assessment cycle.

MRI imaging: approximately 2 h per animal, including anesthesia induction, positioning, scanning, and recovery, for a total of 80 h when all 40 animals are imaged within an assessment cycle. Note that imaging time varies substantially by modality; CT imaging, for example, requires considerably less time per animal than MRI.

Total monthly workload: approximately 192 h or 1.2 FTE. Over the six -month study duration: 7.2 FTE and more than 1,150 total hours.

### Home cage monitoring approach

**Figure 2:**
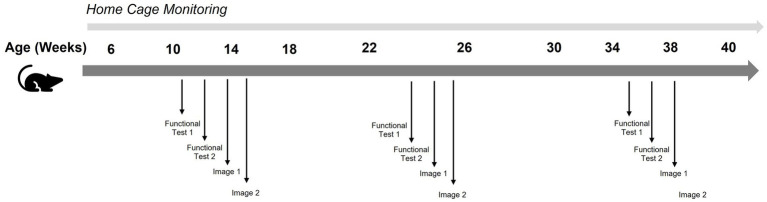
Example of ALS study with home cage monitoring.

With continuous activity monitoring, disease staging happens automatically (see Figure 2). This enables a 50% reduction in functional test frequency, as staging confirmation is derived from home-cage monitoring data. It allows a 50% reduction in imaging sessions because scans target progression points identified by the monitoring system rather than fixed schedules.

Result: approximately 101 h per month or 0.6 FTE. Over 6 months: 3.7 FTE. That is a reduction of 3.5 FTE or 543 labor hours.

### ROI calculation

Assuming a fully loaded technician cost of $50 per hour:

Labor savings per study: 543 h times $50 equals $27,150.

Annual savings assuming 4 similar studies: approximately $108,600.

If the home cage system costs $200,000 with $30,000 annual operating costs:

Payback period equals $200,000 divided by ($108,600 minus $30,000), which equals approximately 2.5 years.

## Technical considerations

### Hardware requirements

Home cage monitoring requires sensors, cameras, data acquisition systems, and storage infrastructure. Multiple hardware components contribute behavioral data: video surveillance provides the richest detail for behavioral phenotyping. RFID supplements visual information with location data, enabling individual animal tracking in group-housed conditions. Capacitance sensors detect activity patterns. Infrared cameras enable dark-phase monitoring. Smart feeding stations capture consummatory behavior. The data acquisition layer synchronizes inputs using high-speed processing units.

Managing the resulting data volume requires robust storage and analytical infrastructure. For context, a facility with 50 monitored cages in our institutional setup can generate approximately 39 GB per hour. This figure is specific to our configuration using high-resolution video at standard frame rates; actual data volumes vary substantially depending on sensor types, video resolution, frame rate, compression algorithms, and the number of simultaneously recorded parameters. Organizations should conduct their own infrastructure assessments during the planning phase. Cloud storage runs $10,000 to $50,000 per year, depending on redundancy requirements. GPU computing for AI analysis adds additional costs.

### Analysis pipelines

Several established tools exist for behavioral analysis. DeepLabCut provides markerless pose estimation (Mathis et al., 2018). SLEAP offers multi-animal tracking capabilities (Pereira et al., 2022). SimBA enables supervised behavioral classification from pose data (Goodwin et al., 2024). For a comprehensive overview of available behavioral analysis tools for home cage monitoring, see Jurek et al. (2026). These open source options complement commercial solutions. The key is building pipelines that reliably convert raw sensor data into meaningful digital biomarkers at scale.

### Data management

FAIR principles matter here: Findable, Accessible, Interoperable, Reusable (Voikar and Gaburro, 2020). Good metadata practices (Moresis et al., 2024) increase the value of collected data and enable cross-study analyses. Organizations that invest in data curation infrastructure will extract more value from their home cage systems over time.

## Barriers to adoption

### Trust and accountability

Digital tools promise precision but face legitimate skepticism. Healthcare and research professionals express valid concerns about AI algorithms. The black box nature of many systems limits the ability to explain and justify decisions. Questions arise about accountability when errors occur. The ethical weight of delegating judgment to automated systems troubles thoughtful practitioners (Panagoulias et al., 2024). These concerns point toward hybrid intelligence approaches that combine human expertise with computational power (Dellermann et al., 2019). The goal is augmentation, not replacement.

### Validation requirements

Performance metrics matter for acceptance. Sensitivity and specificity tell part of the story. In clinical decision support applications, positive and negative predictive values matter more because they indicate the probability that a test result is correct. Scalability ensures systems work across diverse settings. Auditability provides transparency. Without rigorous validation, digital biomarkers will remain research curiosities rather than decision tools.

### Limitations

No technology is perfect. Pretending otherwise does no one any good and undermines credibility.

### Technical limitations

Home cage systems that provide cage-level data cannot distinguish individual animals in group-housed conditions without RFID or visual tagging. This can mask individual variability. Video quality depends on lighting and bedding. Some behavioral constructs require specialized analysis not available in all systems. Species applicability varies. Video tracking works well for primates, while RFID or capacitance sensing may suit rodent applications better.

### Specificity concerns

The specificity limitation applies broadly to many digital biomarkers derived from home cage monitoring, not only to locomotor activity. We have used locomotor activity as a running example throughout this manuscript because it is the most widely measured parameter and therefore serves as an accessible illustration. However, the same principle holds for other metrics: changes in feeding patterns, circadian rhythms, or social interactions can reflect disease progression, treatment effects, stress, environmental disruption, or other factors. Researchers should not over-interpret changes in any single digital biomarker without corroborating evidence. The richness of continuous, multi-parametric data partially compensates for this limitation but does not eliminate it.

### Implementation barriers

Full facility deployment requires substantial capital that not all institutions can access. Implementation demands data science expertise that may not be in-house. IT infrastructure requirements add to the total cost of ownership. Training spans technical operation, data management, and compliance domains. These barriers are real and should factor into planning.

### Regulatory uncertainty

FDA pathways for translational digital biomarkers remain unclear. While the agency has shown interest, there are few approval precedents. This uncertainty affects ROI calculations for pharmaceutical companies hoping to use preclinical digital biomarkers to support clinical development. The landscape will likely evolve, but current ambiguity is a fact. Using translational digital biomarkers that are not FDA-approved can still provide value for internal decision-making when advancing a compound to later clinical phases.

### Author perspective

We should acknowledge our own potential biases. This author group includes representatives from pharmaceutical companies, a CRO, a technology provider, and academia. We believe this diversity strengthens the paper by providing multiple viewpoints. But readers should recognize that our professional interests may influence our perspective. We have tried to present a balanced analysis. Others may weigh the evidence differently.

## Conclusion

The gap between home cage monitoring potential and adoption comes down to measurement. Organizations that cannot quantify value cannot justify investment. This paper provides the missing measurement framework.

We have presented explicit KPIs organized by Scientific, Operational, Welfare, and Financial categories. Each metric has a calculation method and a target benchmark. Different organizations will weigh these factors differently based on their missions and constraints, and that is appropriate. The framework is designed to accommodate varying priorities while maintaining scientific rigor.

The fully digital vivarium remains aspirational for most facilities. Partial implementation, focusing on studies where KPIs show greatest improvement, may represent a pragmatic starting point. As technology costs decline, broader deployment becomes feasible.

The field is evolving rapidly. New technologies integrate at an accelerating pace. Early experience, where possible, positions organizations to make better decisions about future investments. We hope this framework serves as a practical guide for navigating this rapidly evolving landscape.

## Glossary

3Rs: Reduce, refine, replace
AI: Artificial intelligence
ALS: Amyotrophic lateral sclerosis
CRO: Contract Research Organization
CT: Computed tomography
CV: Coefficient of variation
DB: Digital biomarker
DVC®: Digital ventilated cages
ECG: Electrocardiogram
EEG: Electroencephalogram
EMA: European Medicines Agency
FAIR: Findable, accessible, interoperable, reusable
FDA: Food and Drug Administration
FTE: Full-time equivalent
GPU: Graphics processing unit
HCM: Home cage monitoring
Hours_conv_: Hours required under conventional approach
Hours_HCM_: Hours required under home cage monitoring approach
ICH: International Council for Harmonization
IT: Information technology
KPI: Key performance indicator
ML: Machine learning
MRI: Magnetic resonance imaging
*N*_conv_: Sample size under conventional approach
*N*_HCM_: Sample size under home cage monitoring approach
RFID: Radio-frequency identification
ROI: Return on investment
